# The impact of age on rectal cancer treatment, complications and survival

**DOI:** 10.1186/s12885-022-10058-9

**Published:** 2022-09-12

**Authors:** Øystein Høydahl, Tom-Harald Edna, Athanasios Xanthoulis, Stian Lydersen, Birger Henning Endreseth

**Affiliations:** 1 Department of Surgery, Levanger Hospital, Nord-Trøndelag Hospital Trust, Levanger, Norway; 2 IKOM Department of Clinical and Molecular Medicine, NTNU, Norwegian University of Science and Technology, Trondheim, Norway; 3 Regional Centre for Child and Youth Mental Health and Child Welfare – Central Norway, Faculty of Medicine, Department of Mental Health, Faculty of Medicine, Norwegian University of Science and Technology, Trondheim, Norway; 4 Clinic of surgery, St. Olavs Hospital, Trondheim University Hospital, Trondheim, Norway

**Keywords:** Rectal cancer, Elderly, Treatment, Survival, Epidemiology

## Abstract

**Background:**

The number of older patients with rectal cancer is increasing. Treatment outcome discrepancies persist, despite similar treatment guidelines. To offer the oldest patients optimal individually adjusted care, further knowledge is needed regarding treatment strategy and outcome. The present study aimed to evaluate treatment, postoperative complications, and survival in older patients treated for rectal cancer.

**Methods:**

This retrospective study included all 666 patients (*n*=255 females, *n*=411 males) treated for rectal cancer at Levanger Hospital during 1980-2016 (*n*=193 <65 years, *n*=329 65-79 years, *n*=144 ≥80 years). We performed logistic regression to analyse associations between complications, 90-day mortality, and explanatory variables. We performed a relative survival analysis to identify factors associated with short- and long-term survival.

**Results:**

Despite a similar distribution of cancer stages across age-groups, patients aged ≥80 years were treated with a non-curative approach more frequently than younger age groups. Among patients aged ≥80 years, 42% underwent a non-curative treatment approach, compared to 25% of patients aged <65 years, and 25% of patients aged 65-79 years. The 90-day mortality was 15.3% among patients aged ≥80 years, compared to 5.7% among patients aged <65 years, and 9.4% among patients aged 65-79 years.

Among 431 (65%) patients treated with a major resection with curative intent, the 90-day mortality was 5.9% among patients aged ≥80 years (*n*=68), compared to 0.8% among patients aged <65 years (*n*=126), and 3.8% among patients aged 65-79 years (*n*=237). The rate of postoperative complications was 47.6%. Pneumonia was the only complication that occurred more frequently in the older patient group. The severity of complications increased with three factors: age, American Society of Anaesthesiologists score, and >400 ml perioperative blood loss. Among patients that survived the first 90 days, the relative long-term survival rates, five-year local recurrence rates, and metastases rates were independent of age.

**Conclusion:**

Patients aged ≥80 years were less likely to undergo a major resection with curative intent and experienced more severe complications after surgery than patients aged <80 years. When patients aged ≥80 years were treated with a major resection with curative intent, the long-term survival rate was comparable to that of younger patients.

## Introduction

The incidence of rectal cancer in Norway is among the highest in the world [1]. Moreover, the aging of the population has led to a high number of older patients. Questions remain to be resolved regarding rectal cancer treatment in this heterogeneous group of patients to offer optimal individualized care.

Treatments for rectal cancer have evolved over the last four decades. Diagnostic tools have become more available. The diagnostic work-up is performed according to standardized protocols that apply across age-groups [2]. The implementation of total mesorectal excision (TME) and the addition of preoperative radiotherapy for locally advanced tumours have improved survival [3, 4]. Minimally invasive surgery has reduced surgical trauma, and protocols to enhance recovery after surgery have become a standard part of treatment [5, 6]. The modern principles in rectal cancer treatments, including TME and preoperative radiotherapy, have been applied to all patients treated at Levanger Hospital since 1980. A prospective protocol for the operative strategy, radiotherapy, and surveillance was established, and excellent results were reported after the first ten years [7].

The fundamental treatment for rectal cancer includes a resection of the tumour-bearing segment of the rectum. This procedure is associated with substantial postoperative morbidity [8]. Complications may be fatal, particularly in aged, vulnerable patients with low capacity to withstand physiological stress. This risk has had a heavy impact on the choice of treatment for aged patients, and thus, it may adversely affect both functional results and survival. The number of older patients with rectal cancer is expected to increase in the years to come [9]; thus, deeper knowledge is needed to pursue individually optimized care.

The present study aimed to evaluate treatment, complications, and survival in patients with rectal cancer during 1980-2016, with a special focus on the older patients.

## Methods

This study included all patients treated for rectal cancer at Levanger Hospital during 1980-2016. Levanger Hospital was the primary hospital of 10 municipalities in Norway, and the catchment area remained unchanged throughout the study period. The population increased by 18%, from 83,890 inhabitants in 1980, to 99,566 inhabitants in 2016.

We identified patients through the hospital administrative system and reviewed health records for all patients discharged with diagnosis codes for rectal cancer, based on the International Classification of Diseases, 8^th^ revision (ICD-8) codes 154, ICD-9 codes 154, and ICD-10 codes C20. To ensure a complete cohort, the retrieved data were crosschecked and confirmed with data recorded in the Norwegian Cancer Registry during 1980-2016. We retrieved data on demographic variables, comorbidities, treatment, tumour characteristics, histopathology, postoperative complications, and short- and long-term survival.

During the study period, 51 patients in our catchment area were referred for treatment to other hospitals; 33 patients were referred to the nearest university hospital for preoperative radiotherapy and underwent surgery there; and 18 patients chose to receive treatment in another hospital. These patients were not included in our cohort. The characteristics of these patients are presented Table 9 in Appendix. This study included a total of 666 patients treated for rectal cancer.

Rectal cancer was defined as a tumour located within 15 cm of the anal verge, measured with a rigid proctoscope. The rectal sections were defined according to the distance above the anal verge: the proximal rectum was at 12-15 cm; the middle rectum was at 6-11 cm; and the distal rectum was at 0-5 cm.

Disease stages were classified according to the TNM classification, sixth edition [10]. Signs of residual tumour after surgery were classified as R0 - no microscopic residual tumour; R1 - microscopically involved resection margin; and R2 - macroscopic residual tumour. A major resection with curative intent was defined as a resection of the tumour-bearing segment of the rectum, including R1-resections and tumour perforations, with no radiological or preoperative signs of metastases. Major resections were performed according to TME principles [3, 11]. In four patients, the resections were performed laparoscopically, all in the last part of the study period (2010-2016). A histopathological verification of cancer was missing in 20 of 666 patients (3%); however, the rectal cancer diagnosis was evident from other examinations. Among these 20 patients, 7 underwent non-resection procedures, and 13 underwent best supportive care, without resection.

Preoperative radiotherapy was recommended for patients with fixed, locally advanced tumours, according to national guidelines established in 1993 [12]. During 1980-1999, referrals for radiotherapy were based on proctoscopy and digital examinations. In 2000, magnetic resonance imaging of the rectum became available at our hospital, and it was used as the decisive diagnostic modality for evaluating tumour resectability. All patients selected for radiotherapy were referred to the nearest university hospital. The majority of patients received 2 Gy ×25, but in selected cases, patients received 5 Gy ×5.

For this study, patients were categorized into groups according to treatment intent. The curative intent group included patients with (*i*) a major resection (R0 and R1) or (*ii*) a polypectomy. The non-curative intent group included patients with (*iii*) a major resection, (*iv*) a bypass/stoma, or (*v*) best supportive care.

Comorbid conditions were classified according to the American Society of Anaesthesiology (ASA) score and the Charlson Comorbidity Index (CCI) [13, 14]. Emergency surgery was defined as a surgery due to evidence of large bowel obstruction or large bowel perforation.

Postoperative complications included any deviation from the normal postoperative course during the same hospital admission, and were noted in the patient records. The severity of postoperative complications was graded according to the Clavien-Dindo (CD) classification of surgical complications [15].

Clinical follow-up was initially conducted according to local guidelines. Starting in 1993, follow-ups were conducted according to similar national guidelines [2]. Normal follow-ups lasted for 5 years, but they were extended in selected cases. The follow-up period for this study ended on December 31^st^, 2018. The mean follow-up time was 6.12 years (range: 0.02 – 34.04, SD: 5.78).

Survival time calculations started from the date of admission and ended at the last known date that the patient was alive or the date of death. Patients that were alive December 31^st^, 2018, were counted as censored cases. The mean follow-up time with regard to survival was 6.89 years (range: 0.01 - 37.88, SD: 7.49).

### Statistical analysis

The Cochran Armitage exact trend test was performed to test for trends in proportions; for example, the proportion of Hartmann’s procedures (HPs) performed per decennium.

The Joncheere-Terpstra test was performed to test for the distribution of blood loss volumes (dependent variable) across decennium periods (independent variables). Kaplan-Meier analyses were performed to estimate the 5-year rates of local recurrences and metastases.

Logistic regression analysis was performed to test for associations between the 90-day mortality (dependent variable) and different explanatory variables. Ordinal logistic regression was performed to test for associations in doubly ordered r × c contingency tables; for example, the ASA scores in different age groups. The resulting odds ratio (OR) was a common OR estimate for any 2 × 2 contingency table that would occur, if the r ×c table were collapsed to a 2 × 2 table, based on any cut-off threshold, along the columns and rows. Multinomial logistic regression analysis was performed in singly ordered r x c contingency tables; for example, the type of treatment in different age groups.

### Relative survival analysis

Relative survival is a measure of mortality compared to the general population. The observed survival in the group with cancer was divided by the expected survival of a comparable group in the general Norwegian population, matched with respect to age, sex, and the calendar year of investigation. Relative survival was estimated with the Ederer II method and analysed with STATA 16 (StataCorp. 2019. Stata Statistical Software: Release 16. College Station, TX: StataCorp LLC) [16]. Multivariable analyses were performed with a full likelihood approach. We retrieved data on Norwegian population survival probabilities for every year, starting from 1980, calculated for groups divided by sex and age, from the Human Mortality Database [17].

Two-sided *P*-values <0.05 were considered significant. Means are reported with the range (minimum to maximum) and standard deviation (SD), where relevant. Ninety-five percent confidence intervals (CIs) are reported, where relevant. Analyses were performed with STATA 16 (StataCorp. College Station, TX: StataCorp LLC), IBM SPSS Statistics 25 (IBM Corp. Armonk, NY: IBM Corp), and StatXact 9 (Cytel. Waltham, MA).

## Results

### All patients

The characteristics of all 666 patients treated for rectal cancer in 1980-2016 are presented in Table 1. Patients were predominantly male (61.7%), and the mean age was 70.6 years (range: 35.2-97.2, SD: 11.1). Among males, the mean age was 70.3 years (range: 40.7-94.3, SD: 10.3), and among females, the mean age was 71.0 years (range: 35.2-97.1, SD: 12.2). The mean age increased insignificantly from 69.9 years in 1980-1989 to 71.2 years in 2010-2016. The mean number of patients diagnosed with rectal cancer increased from 12.8 patients/year in 1980-1989 to 25.3 patients/year in 2010-2016. We also observed an insignificant increase over time in the proportion of patients aged ≥80 years.

**Table 1:** Characteristics of 666 patients admitted to the hospital with rectal cancer during 1980-2016

Characteristic	Total, n (%)	<65 years old, n (%)	65-79 years old, n (%)	80+ years old, n (%)	*p*
Sex					0.16 ^a^
Female	255 (38)	73 (38)	115 (35)	67 (47)	
Male	411 (62)	120 (62)	214 (65)	77 (53)	
Calendar-year (row %)					0.23^b^
1980-1989	128 (19)	37 (19) (29)	71 (22) (56)	20 (14) (16)	
1990-1999	178 (27)	51 (26) (29)	91 (28) (51)	36 (25) (20)	
2000-2009	183(27)	54 (28) (30)	86 (26) (47)	43 (30) (24)	
2010-2016	177 (27)	51 (26) (29)	81 (25) (46)	45 (31) (25)	
Charlson Comorbidity Index					
0	497 (75)	162 (84)	253 (77)	82 (57)	<0.001^b^
1	70 (11)	18 (9)	38 (11)	14 (10)	
2 +	99 (15)	13 (7)	38 (11)	48 (33)	
ASA score					<0.001^b^
1-2	402 (60)	160 (83)	204 (62)	38 (26)	
3	235 (35)	30 (16)	115 (35)	90 (63)	
4-5	29 (4)	3 (2)	10 (3)	16 (11)	
Localization (distance proximal to the anal verge)					0.006 ^b^
Proximal (12-15 cm)	210 (32)	71 (37)	102 (31)	37 (26)	
Middle (6-11 cm)	280 (42)	81 (42)	140 (42)	59 (41)	
Distal (0-5 cm)	176 (26)	41 (21)	87 (27)	48 (33)	
Stage (TNM)					0.89^c^
I	150 (23)	49 (25)	73 (22)	28 (19)	
II	195 (29)	51 (26)	110 (33)	34 (24)	
III	153 (23)	43 (22)	77 (23)	33 (23)	
IV	124 (19)	42 (22)	58 (18)	24 (17)	
Unknown	44 (7)	8 (4)	11 (3)	25 (17)	
Treatment intent categories					<0.001^d^
Curative intent					
Major resection	433 (65)	127 (66)	238 (72)	68 (47)	
Polypectomy	41 (6)	18 (9)	8 (2)	15 (10)	
Non-curative intent					
Major resection	58 (9)	24 (12)	27 (8)	7 (5)	
Bypass/Stoma	47 (7)	9 (5)	22 (7)	16 (11)	
Best supportive care ^f^	87 (13)	15 (8)	34 (10)	38 (26)	
Surgery					0.68^a^
Elective surgery ^g^	551 (95)	169 (95)	280 (95)	102 (96)	
Emergency surgery	28 (5)	9 (5)	15 (5)	4 (4)	

^a^ Cochran-Armitage exact trend test

^b^ Ordinal logistic regression with the age group as a covariate

^c^ Ordinal logistic regression with the age group as a covariate, for known stages

^d^ Multinomial logistic regression with the age group as a covariate

^e^ Palliative surgery (stoma, by-pass, palliative resection)

^f^ Including palliative radiochemotherapy in 3 cases

^g^ Including polypectomy

The CCI and ASA score increased with increasing age. Distal tumours were more prevalent in the oldest age group. The rate of patients with stages I and II tumours increased throughout the study period, but the rate of patients with unknown stages declined; only one patient had an unknown stage in the last time period (2010-2016). Overall, 17% of patients aged ≥80 years had an unknown tumour stage. This rate declined from 41.7% in 1990-1999 to 0% in 2010-2016. The distribution of tumour stages did not differ between age groups.

The overall rate of patients treated with a major resection with curative intent was 65%, and this rate remained consistent throughout the study period. The rate varied across age-groups; it was 65% among patients under 65 years old, 72% among patients 65-79 years old, and 47% among patients ≥80 years old. The distribution of treatment intent categories differed across age groups. The proportion of patients that underwent best supportive care was higher among patients ≥80 years old.

Among patients treated with a non-curative intent, 27.1% (52/192) received chemotherapy. Chemotherapy was performed in 54.2% (26/48) of patients <65 years old, 30.1% (25/83) of patients 65-79 years old, and 1.6% (1/61) of patients ≥80 years old. In examining different time periods, we found that chemotherapy was performed in 36.1% (13/36) of patients during 1980-1989, 13.8% (8/58) during 1990-1999, 34% (17/50) during 2000-2009, and 29.2% (14/48) during 2010-2016.

Radiotherapy was administered to 33% (63/192) of the patients in the non-curative treatment intent group. Among these, 13 patients underwent radiotherapy as a part of a curative treatment plan, and 50 patients underwent palliative radiotherapy. Among the patients treated with palliative radiotherapy, 29 had metastases at diagnosis.

The 90-day mortality, overall survival, and relative long-term survival rates for all patients are presented in Table 2. The 90-day mortality after admission was 9.6%, and it increased significantly with age. In addition, the five-year overall and relative survival rates in patients that survived the first 90 days decreased with age. Prognostic factors associated with 90-day mortality are presented in Table 3. Age was not a significant factor, but the calendar year of treatment, a high ASA score, and the treatment intent category were significant independent variables. The five-year relative survival rates among all patients were 62.6% (95% CI: 51.2 to 73.1) during 1980-1989, 48.9% (95% CI: 40.1 to 57.6) during 1990-1999, 61.4% (95% CI: 52.3 to 69.9) during 2000-2009, and 67.6% (95% CI: 58.1 to 76.3) during 2010-2016.

**Table 2:** Analysis of 90-day mortality, five-year overall survival, and five-year relative survival, according to age group

Age group	Patients	Death within 90 days	Patients that survived 90 days (602 patients)	
Years	N	N/total, (%)	Overall survival	Relative survival
		*p*=0.004 ^a^	% (95% CI) *p*<0.001^b^	% (95% CI) *p*<0.001^c^
<65	193	11 / 193 (5.7)	69.9 (63.1 to 76.7)	72.9 (65.3 to 79.3)
65 - 79	329	31 / 329 (9.4)	56.0 (50.2 to 61.8)	66.9 (59.9 to 73.5)
80 +	144	22 / 144 (15.3)	25.6 (17.6 to 33.6)	48.3 (34.2 to 63.6)
Total	666	64 / 666 (9.6)	54.0(49.8 to 58.2)	66.3 (61.2 to 71.1)

^a^Cochran Armitage exact trend test

^b^Log Rank test

^c^Log likelihood

**Table 3:** Logistic regression results identified factors associated with death within 90 days for all patients diagnosed with rectal cancer in 1980-2016

Factor	Death within 90 days, N/total	Unadjusted odds ratio (95% CI)	*p* value	Adjusted odds ratio (95% CI)	*p* value
Age (years)					
<65	11/193 (7%)	1 (reference)		1 (reference)	
65 - 79	32/329 (9.7%)	1.78 (0.88 to 3.62)	0.11	1.31 (0.58 to 2.97)	0.51
80+	22/144 (15.3%)	2.98 (1.40 to 6.37)	0.005	1.12 (0.43 to 2.91)	0.82
Calendar year		0.96 (0.94 to 0.99)	0.003	0.94 (0.91 to 0.97)	<0.001
ASA score					<0.001
1-2	23/402 (5.7%)	1 (reference)		1 (reference)	
3	28/235 (11.9%)	2.23 (1.25 to 3.97)	0.006	1.64 (0.81 to 3.29)	0.17
4-5	14/29 (48.3%)	15.38 (6.63 to 35.67)	<0.001	4.18 (1.40 to 12.50)	0.01
Emergency surgery	4/27 (14.8%)	1.68 (0.56 to 5.01)	0.36	1.04 (0.27 to 4.05)	0.95
Treatment intent categories					
Curative intent					
Major resection	14/433 (3.2%)	1 (reference)		1 (reference)	
Polypectomy	0/41	1		1	
Non-curative intent					
Major resection	5/58 (8.6%)	2.82 (0.98 to 8.15)	0.055	2.16 (0.73 to 6.4)	0.16
Bypass, stoma	14/47 (29.8%)	12.70 (5.59 to 28.86)	<0.001	11.11 (4.46 to 27.66)	<0.001
Best supportive care	32/87 (36.8%)	17.41 (8.75 to 34.65)	<0.001	15.99 (7.21 to 35.44	<0.001

Logistic regression was performed with death within 90 days as the dependent variable. Unadjusted was performed with one covariate at a time; adjusted was performed with all the listed covariates simultaneously

### Patients with stages I-III disease treated with a major resection with curative intent

The characteristics of 431 (64.7%) patients with rectal cancer stages I-III that were treated with a major resection with curative intent (R0 and R1) are presented in Table 4. These patients were predominantly males (63.1%). The mean age remained stable during the study period; the mean ages were 69.1 (range: 40.7-91.8, SD: 9.7) years in males and 69.8 (range: 37.4-91.6, SD: 11.5) years in females. The mean annual number of patients that underwent a major resection with curative intent doubled over time, from 8.2 patients/year in 1980-1989 to 16.3 patients/year in 2010-2016. Tumour stages, tumour localizations, and the use of radiotherapy were equally distributed across the age groups. CCI and ASA scores increased with age. Older patients less often underwent an anterior resection or an abdominoperineal resection, and more often underwent an HP, compared to younger patients. The rate of HPs decreased from 3.7% (3/82) during 1980-1989 to 2.6% (3/116) during 1990-1999; thereafter, the rate increased to 14.3% (17/119) during 2000-2009 and to 22.8% (26/114) during 2010-2016 (*p*<0.001).

**Table 4:** Characteristics of 431 patients with stages I-III rectal cancer that underwent a major resection with curative intent in 1980-2016, grouped according to age

Characteristic	Total n (%)	Age <65 years, n (%)	Age 65-79 years, n (%)	Age 80+ years, n (%)	*P-*value
Proportion of total	431/666 (65)	126/193 (65)	237/328 (72)	68/144 (47%)	
Sex					0.29 ^a^
Females	159 (37)	46 (37)	81 (34)	32 (47)	
Males	272 (63)	80 (63)	156 (65)	36 (53)	
Calendar-year (row %)					0.50 ^b^
1980-1989	82 (19)	23 (28)	49 (60)	10 (12)	
1990-1999	116 (27)	31 (27)	71 (62)	14 (12)	
2000-2009	119 (28)	38 (32)	60 (50)	21 (18)	
2010-2016	114 (26)	34 (30)	57 (50)	23 (20)	
Charlson Comorbidity Index					0.001 ^b^
0	350 (81)	114 (90)	187 (79)	49 (72)	
1	35 (8)	7 (6)	22 (9)	6 (9)	
2 +	46 (11)	5 (4)	28 (12)	13 (19)	
ASA score					<0.001 ^b^
1-2	291 (68)	108 (86)	157 (66)	26 (38)	
3	138 (32)	18 (14)	78 (33)	42 (62)	
4-5	2 (0.5)	0	2 (1)	0	
Localization in rectum					0.25 ^b^
Proximal	153 (36)	51 (40)	79 (33)	23 (34)	
Middle	178 (41)	50 (40)	99 (42)	29 (43)	
Distal	100 (23)	25 (20)	59 (25)	16 (24)	
Stage (TNM)					0.16 ^b^
I	119 (28)	38 (30)	66 (28)	15 (22)	
II	173 (40)	49 (39)	99 (42)	25 (37)	
III	139 (32)	39 (31)	72 (30)	28 (41)	
Radiochemotherapy					0.12 ^c^
No	345 (80)	96 (76)	190 (80)	59 (87)	
Preoperatively	71 (16)	23 (18)	39 (16)	9 (13)	
Postoperatively	14 (3)	7 (6)	7 (3)	0	
Both pre- and postoperatively	1 (0.2)	0	1 (0.4)	0	
Treatment					<0.001 ^c^
AR	276 (64)	89 (71)	155 (65)	32 (47)	
APR	104 (24)	30 (24)	62 (26)	12 (18)	
Hartmann’s procedure	48 (11)	7 (6)	18 (8)	23 (34)	
Proctocolectomy	3 (1)	0	2 (1)	1 (1)	
Surgery					0.33 ^a^
Elective surgery	421 (98)	125 (99)	230 (97)	66 (97)	
Emergency surgery	10 (2)	1 (1)	7 (3)	2 (3)	
R stage					0.42 ^b^
R0	405 (94)	121 (96)	222 (94)	62 (91)	
R0 with perforation	14 (3)	3 (2)	7 (3)	4 (6)	
R1	12 (3)	2 (2)	8 (2)	2 (3)	

*AR* anterior resection, *APR* abdominoperineal resection

^a^ Cochran-Armitage exact trend test

^b^ Ordinal logistic regression with age group as a covariate

^c^ Multinomial logistic regression with age group as a covariate

The proportion of patients with CCI scores ≥2 increased steadily over time. The proportions were 7.3% (6/82) in 1980-1989, 12.1% (14/116) in 1990-1999, 7.6% (9/119) in 2000-2009, and 14.9% (17/114) in 2010-2016. The proportion of patients with ASA scores >2 also increased throughout the observational period. The proportions were 19.5% (16/82) in 1980-1989, 36.2% (42/116) in 1990-1999, 28.6% (34/119) in 2000-2009, and 42.1% (48/114) in 2010-2016 (*p*=0.008).

Preoperative radiotherapy was administered to 7.3% (6/82) of patients during 1980-1989, 0.9% (1/116) of patients during 1990-1999, 26.1% (31/119) of patients during 2000-2009, and 29.8% (34/114) of patients during 2010-2016 (*p*<0.001).

### Postoperative complications

Major complications (CD ≥3) occurred in 13.5% (58/431) of all patients; they occurred in 10.3% (13/126) of patients aged ≤65 years, 14.4% (34/236) of patients aged 65-79 years, and 15.9% (11/69) of patients aged ≥80 years (*p*=0.24). The proportion of patients with major complications increased from 11.0% (9/82) during 1980-1989, to 13.8% (16/116) during 1990-1999, then decreased to 10.9% (13/119) during 2000-2009, and then increased to 21.1% (24/114) during 2010-2016 (*p*=0.035). An anastomotic leak was diagnosed in 4.9% (21/431) of patients, and wound dehiscence was diagnosed in 1.9% (8/431) of patients.

Infective complications occurred in 35.7% (154/431) of patients that underwent a major resection with a curative intent. The most common infective complications were urinary tract infections (18.6%, *n*=80/431), wound infections (10.7%, *n*=46/431), intra-abdominal abscesses (5.6%, *n*=24/431), and pneumonia (3.5%, *n*=15/431). Pneumonia was the only complication that occurred significantly more frequently in the oldest group (≥80 years: 8.8%, *n*=6/68) compared to younger patients (<80 years: 2.5%, *n*=9/363; *p*=0.015).

Blood loss declined in each decade; the mean blood loss volumes were 1388 ml (range: 300-9000, SD: 1182) during 1980-1989, 1216 ml (range 200-10000, SD: 1234) during 1990-1999, 732 ml (range: 50-3300, SD: 647) during 2000-2009, and 427 ml (range 0-2500, SD: 328) during 2010-2016 (*p*<0.001). Blood transfusions were administered to 87.8% (72/82) of patients in 1980-1989, compared to 26.3% (30/114) of patients in 2010-2016.

A reoperation (CD ≥3b) was required in 11.4% (49/431) of all patients that underwent a major resection. The frequency of reoperations increased during the last part of the study period; it was 7.3% (6/82) during 1980-1989, 9.5% (11/116) during 1990-1999, and 9.2% (11/119) during 2000-2009, but increased to 18.4% (21/114) during 2010-2016 (*p*=0.037). Reoperations were performed in 8.8% (6/68) of patients aged ≥80 years and in 11.9% (43/363) of patients aged <80 years (*p*=0.60).

Ordinal multivariable logistic regression analyses of risk factors associated with the CD severity of postoperative complications are presented in Table 5. Independent risk factors were: increasing age, increasing ASA scores, and perioperative blood loss >400 ml.

**Table 5:** Factors associated with postoperative complications, based on Clavien-Dindo scores, in 431 patients treated for Stages I-III rectal cancer with a major resection with curative intent (R0 and R1)

Factor	Unadjusted odds ratio (95% CI)	*p* value	Adjusted odds ratio (95% CI)	*p* value
Age (years)				
<65	1 (reference)		1 (reference)	
65 - 79	2.07 (1.36 to 3.14)	0.001	1.91 (1.22 to 2.99)	0.005
80 +	2.22 (1.26 to 3.90)	0.005	2.20 (1.15 to 4.22)	0.017
Female sex	0.83 (0.57 to 1.19)	0.31	0.96 (0.65 to 1.41)	0.82
Calendar-year				
1980-1989	1 (reference)		1 (reference)	
1990-1999	0.46 (0.27 to 0.79)	0.004	0.49 (0.28 to 0.87)	0.014
2000-2009	0.34 (0.20 to 0.58)	<0.001	0.56 (0.30 to 1.02)	0.059
2010-2016	0.44 (0.26 to 0.75)	0.003	1.12 (0.56 to 2.25)	0.75
Preoperative radiochemotherapy	1.11 (0.68 to 1.80)	0.68	0.98 (0.56 to 1.71)	0.93
ASA score				
1–2	1 (reference)		1 (reference)	
3	1.80 (1.23 to 2.65)	0.003	1.74 (1.14 to 2.68)	0.011
4	20.99 (1.05 to 421)	0.047	11.04 (0.70 to 173)	0.087
Treatment				
AR	1 (reference)		1 (reference)	
APR	2.05 (1.33 to 3.14)	0.001	1.25 (0.77 to 2.00)	0.37
Hartmann’s procedure	1.52 (0.86 to 2.66)	0.15	1.11 (0.55 to 1.71)	0.76
Emergency surgery	1.16 (0.39 to 3.47)	0.79	0.44 (0.13 to 1.56)	0.21
Surgery (duration in min)				
<90	1 (reference)		1 (reference)	
90-179	2.15 (0.98 to 6.47)	0.55	1.50 (0.53 to 4.20)	0.44
180 +	6.76 (2.58 to 17.7)	<0.001	2.41 (0.78 to 7.46)	0.13
Blood loss (ml)				
0-200	1 (reference)		1 (reference)	
201-400	0.94 (0.39 to 2.26)	0.90	1.04 (0.41 to 2.65)	0.93
401-800	2.73 (1.20 to 6.22)	0.017	2.59 (1.03 to 6.53)	0.043
>800	5.60 (2.47 to 12.69)	<0.001	5.37 (2.01 to 14.33)	0.001

Ordinal multivariable logistic regression analysis was performed with the Clavien-Dindo score as the dependent variable. Unadjusted: performed with one covariate at a time; adjusted: performed with all the listed covariates simultaneously

### Short- and long-term survival among patients that underwent a major resection with curative intent

The 90-day mortality, overall survival, and relative long-term survival rates in patients with rectal cancer stage I-III that underwent a major resection with curative intent are presented in Table 6. The 90-day mortality rate after admission was 3.2%.

**Table 6:** Rates of 90-day mortality, five-year overall survival, and five-year relative survival, according to age ^*^

Age group	Patients	Death within 90 days	Patients that survived 90 days (417 patients)	
Years	N	N/total (%)	Overall survival	Relative survival
		*p*=0.061 ^a^	% (95% CI) *p*<0.001 ^b^	% (95% CI) *p*=0.12 ^c^
<65	126	1 / 126 (0.8)	86.8 (80.6 to 93.0)	90.5 (82.8 to 95.6)
65-79	237	9 / 237 (3.8)	67.9 (61.7 to 74.0)	81.4 (75.6 to 88.2)
80+	68	4 / 68 (5.9)	40.8 (28.5 to 52.7)	74.2 (51.9 to 95.9)
Total	431	14 / 431 (3.2)	69.4 (64.7 to 73.6)	84.2 (78.5 to 89.3)

^a^Cochran Armitage exact trend test

^b^Log Rank test

^c^Log likelihood

^*^This analysis included patients with rectal cancer stages I-III that underwent a major resection with curative intent

The five-year overall survival rates decreased significantly with age. The 10-year, 20-year, and 30-year estimated survival rates were 51.6% (95% CI: 46.4 to 56.8), 27.4% (95% CI: 21.8 to 33.0), and 7.9% (95% CI: 2.9 to 12.9), respectively. The mean survival time was 13.2 years (95% CI: 12.0 to 14.4).

**Fig. 1:**
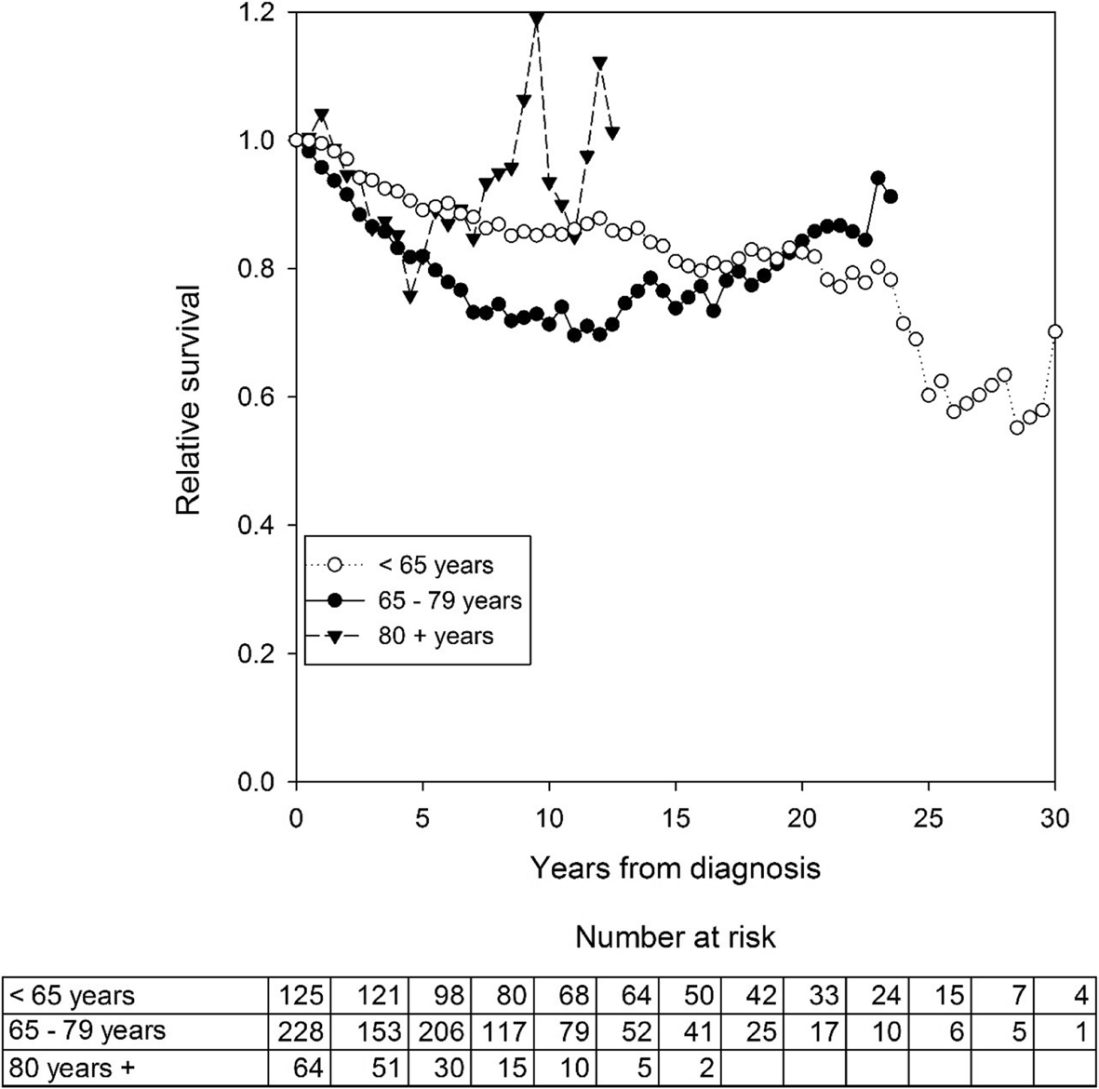
Relative survival after resection with curative intent among patients that survived 90 days (*N*=417) in different age groups. Each column represents 2.5 years.

The five-year relative survival rates decreased insignificantly with age in this patient group (Table 6). The 10-year, 20-year, and 30-year relative survival rates were 79.6% (95% CI: 71.5 to 87.3), 82.6% (95% CI: 66.6 to 99.4), and 73.1% (95% CI: 35.9 to 128), respectively. Relative survival rates in patients that survived 90 days after a major resection with curative intent varied with the age-group (Figure 1). The five-year relative survival rates in patients that survived the first 90 days also varied over time; they were 89.4% (95% CI: 74.8 to 100) during 1980-1989, 72.0% (95% CI: 60.3 to 82.2) during 1990-1999, 87.1% (95% CI: 76.1 to 95.7) during 2000-2009, and 90.4% (95% CI: 78.9 to 99.0) during 2010-2016.

The five-year relative survival rates depended on the type of resection. At five years after R0 resections, R0 resections with a tumour perforation, or R1 resections, survival rates were 86.4% (95% CI: 80.5 to 91.5), 57.1% (95% CI: 22.2 to 88.3), and 34.8% (95% CI: 8.3 to 66.5), respectively.

Multivariable analyses identified several factors associated with 90-day mortality (Table 7). Mortality increased with increasing age and ASA scores, and decreased over time (i.e., calendar year).

**Table 7:** Factors associated with death within 90 days, in 431 patients treated for rectal cancer stages I-III with a major resection with curative intent (R0 or R1 resection) in 1980-2016

Factor	Unadjusted odds ratio (95% CI)	*p* value	Adjusted odds ratio (95% CI)	*p* value
Age (years)	1.08 (1.02 to 1.16)	0.011	1.08 (1.004 to 1.16)	0.036
Calendar year	0.94 (0.89 to 0.99)	0.023	0.92 (0.87 to 0.98)	0.006
ASA score ^a^	3.43 (1.24 to 9.48)	0.018	3.33 (1.08 to 10.31)	0.037

Multivariable logistic regression analysis was performed with death as the dependent variable. Unadjusted: performed with one covariate at a time; adjusted: performed with all the listed covariates simultaneously

^a^ ASA scores were compared between the following groups: 1-2, 3, 4-5

Prognostic factors associated with long-term relative survival are presented in Table 8. Age was not significantly associated with relative survival. However, CCIs ≥3, increasing ASA scores, emergency surgeries, and stage III disease were significantly inversely associated with long-term survival.

**Table 8:** Factors associated with long-term relative survival in 417 patients treated for rectal cancer stages I-III with a major resection and curative intent (R0 and R1) that survived 90 days postoperatively *(270 died during the observation period)*

Factor	Unadjusted hazard ratio (95% CI)	*p* value	Adjusted hazard ratio (95% CI)	*p* value
Age (years)				
<65	1 (reference)		1 (reference)	
65 - 79	2.25 (0.95 to 5.32)	0.065	1.82 (0.71 to 4.67)	0.22
80 +	2.92 (0.86 to 9.77)	0.081	1.58 (0.42 to 5.93)	0.50
Female sex	1.24 (0.60 to 2.56)	0.57	1.17 (0.60 to 2.29)	0.65
Calendar year				
1980-1989	1 (reference)		1 (reference)	
1990-1999	1.63 (0.60 to 4.43)	0.34	0.95 (0.35 to 2.60)	0.92
2000-2009	1.01 (0.36 to 2.87)	0.98	0.74 (0.27 to 2.05)	0.57
2010-2016	0.52 (0.13 to 2.13)	0.36	0.42 (0.13 to 1.34)	0.14
Charlson Index				
0	1 (reference)		1 (reference)	
1	2.97 (1.14 to 7.74)	0.026	2.06 (0.83 to 5.12)	0.12
2	4.09 (1.71 to 9.78)	0.002	1.78 (0.66 to 4.82)	0.26
3 +	6.08 (1.91 to 19.31)	0.002	4.90 (1.47 to 16.30)	0.010
ASA score				
1-2	1 (reference)		1 (reference)	
3-4	3.43 (1.70 to 6.91)	0.001	2.37 (1.11 to 5.05)	0.026
Emergency surgery	7.19 (2.63 to 19.64)	<0.001	4.88 (1.23 to 19.41)	0.025
TNM-stage				
I-II	1 (reference)		1 (reference)	
III	3.72 (1.67 to 8.29)	0.001	2.70 (1.32 to 5.52)	0.007
Type of resection				
R_0_ - resection	1 (reference)		1 (reference)	
R_0_ - resection with perforation	2.92 (0.78 to 10.94)	0.11	1.20 (0.23 to 6.21)	0.83
R_1_ - resection	5.98 (2.26 to 15.84)	<0.001	2.03 (0.65 to 6.37)	0.23

Unadjusted: performed with one covariate at a time; adjusted: performed with all the listed covariates simultaneously

### Local recurrence and metastasis among patients that underwent a major resection with curative intent

Local recurrence was diagnosed in 7% (29/417) of patients with rectal cancer stage I-III that underwent a major resection with curative intent. The overall estimated five-year local recurrence rate was 7.3% (95% CI: 4.5 to 10.1). The estimated five-year local recurrence rates after an R0 resection, an R0 resection with tumour perforation, and an R1 resection were 4.9% (95% CI: 2.5 to 7.3), 29.7% (95% CI: 0.1 to 59.3), and 78.8% (95% CI: 52.4 to 100), respectively. The five-year local recurrence rates varied by the decade of treatment; they were 4.4% (95% CI: 0 to 9.4) during 1980-1989, 18.5% (95% CI:10.3 to 26.7) during 1990-1999, 2.1% (95% CI: 0 to 5.1) during 2000-2009, and 5.9% (95% CI: 0.7 to 11.1) during 2010-2016 (*p*<0.001). The estimated five-year local recurrence rates were not affected by age.

Metachronous metastases were diagnosed in 21.8% (91/417) of patients. The overall estimated five-year metastasis rate was 22.6% (95% CI: 18.2 to 27.0). The estimated five-year metastasis rates after an R0 resection, an R0 resection with tumour perforation, and an R1 resection were 19.5% (95% CI: 15.1 to 23.9), 58.3% (95% CI: 29.7 to 86.9), and 86.4% (95% CI: 61.6 to 100), respectively. The estimated five-year metastasis rates did not vary significantly by the treatment decade or patient age.

## Discussion

The present study showed that the TNM stage at presentation was equally distributed across age groups. The overall rate of patients treated with a major resection with curative intent was 65%, but the rate varied across age groups: it was 47% among patients aged ≥80 years. One or more postoperative complications occurred in 47.6% of patients. The rates of postoperative complications were independent of age, except for pneumonia, which was more common in patients aged ≥80 years. The severity of postoperative complications, based on the CD score, increased with patient age, ASA score, and perioperative blood loss. The 90-day mortality rate was 3.2%, and the rate increased with age: it was 5.9% among patients aged ≥80 years. In patients that survived the first 90 days, the rates of five-year relative survival, local recurrences, and metastases were independent of age.

### All patients

The incidence of rectal cancer has increased since the 1980s, at both the global and national levels. The main reasons for this increase are an increasing human development index [18], an aging population [19], and an age-independent approach to the diagnostic work-up of suspected cancer. We observed a successive increase in the rectal cancer incidence during the study period and a trend towards an increase in the rate of patients aged ≥80 years. Despite scarce evidence and a demand for knowledge, older patients are frequently excluded from clinical trials [20]. The present study included an unselected consecutive series of all patients treated for rectal cancer at a local hospital during nearly four decades, with a focus on patients aged ≥80 years.

It has been well documented that inequities concerning rectal cancer treatment occur across age groups [21]. The optimal treatment for an individual patient is based on a complete staging of the disease. In the present series, the rate of patients with an unknown stage declined over time, and it was low compared to other series [22]. Tumour stages were evenly distributed across age groups, consistent with previous reports [23]. Although the disease stage is typically the defining determinant in treating younger patients, factors associated with increasing age highly influence treatment options in older patients [24].

In Norway, a standardized diagnostic work-up applies to all patients with rectal cancer [2]. It culminates in a summary meeting of a multidisciplinary team (MDT), where treatment options are considered in detail, based on diagnostic findings and the defined stage of disease. A thorough, objective evaluation of the patient’s functional and physiological status and the patient’s personal preferences regarding treatment are not emphasized in routine care; however, adding these features to routine care would constitute a major improvement in guidance for making decisions for these patients [25].

A non-curative treatment approach was applied to 28.7% of the patients in this study, consistent with previously reported 25-30% rates for incurable disease at diagnosis [1]. Despite a similar stage distribution between age groups at diagnosis, the rate of patients that underwent a non-curative treatment increased with age. Among patients aged ≥80 years, 42% underwent non-curative treatments. Only 17% of the older patients had verified stage IV disease at diagnosis, but 25% underwent non-curative treatment with an unknown stage of disease or a potentially resectable disease. Limitations regarding treatment in older patients are related to the coinciding peak incidences of co-morbid diseases, cognitive impairments, and physical impairments [26]. In the present study, objective measures of co-morbidity, ASA, and CCI scores increased significantly with age.

Palliative resection procedures were more common in younger age groups; the older patients more frequently underwent best supportive care. The overall rate of chemotherapy was 27.1%, and it declined substantially with patient age. Individualized treatment regimens may be well tolerated in older patients with good performance status, hence chronological age should not preclude these patients from chemotherapy [27, 28].

The overall rate of procedures with curative intent was 71.3%. The rate of major resections was 65.1% and the rate of polypectomies was 6.2%. These rates were comparable to the major resection rates of 59.9-70.8% reported recently in an evaluation of Scandinavian and English patients with rectal cancer during 2010-2012 [22]. The present study found a resection rate of 66.3% in our Norwegian population. Resection rates decline consistently with increasing age, and they have varied substantially between countries, despite comparable treatment guidelines. In the present study, 47.2% of patients aged ≥80 years underwent a major resection with curative intent. In comparison, Swedish patients aged >75 years had resection rates of 61.9%, and English patients aged >75 years had resection rates of 45.7%.

The overall 90-day mortality rate was 9.6%. It increased with age, but decreased significantly throughout the study period. Among patients that survived the first 90 days in this series, the five-year relative survival rate was 66.3%. The rate decreased from 72.9% in patients aged <65 years to 48.3% in patients aged ≥80 years. The overall five-year relative survival rates for Norwegian patients with rectal cancer have increased successively over the years, from 43.8% during 1980-1984 to 72.4% during 2016-2020 [1, 29]. Comparable rates during 2012-2016 have been reported in the other Nordic countries and the Netherlands [30, 31].

Selecting the appropriate individualized treatment for rectal cancer is a major challenge in efforts to reduce morbidity and increase survival. The adverse effects of over-treatment may cause unnecessary harm, but under-treatment may reduce survival. Older patients with reduced physiological reserves are particularly prone to the adverse effects of cancer treatment, regardless of whether the approach is curative. This dilemma is reflected by differences in treatment rates, and it underlines the need for additional improvements in the treatment selection process for this group of patients.

### Patients with stages I-III disease treated with a major resection with curative intent

Overall, an anterior resection was the most common procedure, with a rate of 64%. Patients aged ≥80 years had lower anterior resection rates (47%) and were more frequently treated with an HP (34%). This observation was consistent with previous findings [32, 33]. The main advantage of a HP is that it avoids an anastomosis, which eliminates the potentially fatal effects of an anastomotic leak. Among older patients with reduced tolerability for surgical complications, the HP stands out as a safe choice. In this series, only three patients underwent surgery with a laparoscopic approach. Minimally invasive surgery should be considered for older patients as previous studies have demonstrated comparable postoperative outcomes as in younger patients [34].

A Swedish study that examined the postoperative outcome of an HP for rectal cancer found an overall HP rate identical to that found in the present study (11%). They reported that the HP was performed predominantly in older patients (mean age 79 years) with increased co-morbidities, elevated ASA scores, and a poor WHO performance status [35]. In fragile patients, HP has the benefits of a shorter operative time, less bleeding, and a lower rate of serious complications, compared to other treatments. However, we lack evidence that clearly favours either the anterior resection or the HP [36]. A substantial number of patients that undergo anterior resections experience low anterior resection syndrome (LARS) [37]. However, the adverse effects of an end-colostomy due to a HP are well-documented [38].

Throughout the study period, in older individuals, the procedure of choice was increasingly an HP. The frequency of HPs increased from 3.7%, in the first observation period, to 22.8% in the last observation period. The increasing use of an HP over time was also observed previously by other authors [39, 40]. The increasing proportion of older patients with comorbidities over time in our cohort might partly explain this observation. However, because the HP rate increased faster than the increasing proportion of older patients over time, our findings also indicated that there was a general trend towards an increased use of HP.

Following rectal cancer surgery, older patients are encumbered with considerable morbidity, ranging from acute infectious complications to permanent functional derangements [41]. Nevertheless, a limited number of studies have addressed complications in this group of patients [42]. Complication rates depend on patient selection for the study, the rate of emergency surgery, the stage of disease, and the level of the institution. The rate of complications in the present study was considerable (47.6%). Although high, this rate was comparable to rates found in previous studies (21-61%) [32, 43]. Rates of major complications (CD ≥3) and anastomotic leaks were also comparable to those found in previous studies [42]. Complication rates did not differ significantly between patients under and over age 80 years, except for pneumonia, which was more common in older patients. Measures to prevent pneumonia in patients that require surgery have been shown to be effective [44], and should be considered routine care in older patients that undergo rectal cancer surgery. The elevated rate of severe complications that we observed in older patients highlighted their reduced capacity to withstand adverse postoperative events.

The rate of reoperations increased significantly during the study period, from 7.3% during 1980-1989 to 18.4% during 2010-2016. In comparison, a 2011 report of nearly 250,000 English patients observed a reoperation rate of 7.4% [45]. The increasing number of older patients with high ASA categories in recent years might have contributed to this observation. The increasing use of HP was likely an attempt to counterbalance the risk of severe complications that might require reoperations. The number of surgeons that performed rectal cancer surgeries increased throughout the study period; this factor may have adversely impacted the rate of postoperative morbidity, due to the complexity of these procedures. Rectal cancer surgery should be applied by highly experienced teams and in concordance with the latest knowledge. Previous studies that evaluated associations between complication rates and treatment volumes have shown conflicting results [46, 47].

The 90-day mortality in patients aged ≥80 years that underwent a major resection with curative intent was 5.9%, compared to an overall 90-day mortality of 3.2%. These rates were low, compared to rates reported previously [48, 49]. These relatively low rates could indicate that the selection of individuals fit for surgery was appropriate in this series. The five-year relative survival rate for all patients that underwent a major resection with curative intent was 84.2%. In comparison, the Norwegian national relative five-year survival rates for localized, regional, and metastasized rectal cancer during 2016-2020 were 98.2%, 81.5%, and 22.4%, respectively [1]. In contrast, the relative survival for patients that underwent surgery for stages I-III disease was 88.5% during 2016-2020 [50]. Previous reports that compared relative survival across age groups of patients treated with curative intent have shown acceptable long-term survival rates among older patients [32, 51–53]. Therefore, resection surgery should not be withheld based on chronological age.

In the present study, the overall five-year local recurrence rate was 7.3%. During the 1990s, treatment guideline violations, reflected by a low (0.9%) rate of preoperative radiotherapy, resulted in a high local recurrence rate (18.5%) and an adverse relative survival rate (72.0%) for that period [4]. During the two later time-periods, local recurrence rates declined in parallel with an increase in relative survival, as the rate of preoperative radiotherapy increased [54]. The increasing use of radiotherapy observed in the present study was also observed at a national level [55]. The estimated five-year metastasis rate after an R0 resection was 19.5%. This rate was comparable to the national rates (20.2-22.1%) during 2006-2020, for patients that underwent resections for stages I-III disease [50].

### Strengths and weaknesses

The main strength of the present study was the inclusion of a large number of consecutive patients treated for rectal cancer at a local hospital, in accordance with evidence-based guidelines. Another strength was the long-term observation period of 37 years. Our institution was the primary hospital for a stable population throughout the observational period, and the population was suitable for evaluating trends over time [56]. Complications for each patient were retrieved from hospital records. Norwegian referral policies are practically age-independent; hence, we believe only a small number of patients was not included in the scope of the current report.

The main limitation of the study was its retrospective design. Unknown or unrecorded confounders might have affected decisions regarding patient selection and treatment. An unknown number of complications may have gone unnoticed, and thus, were not included in the hospital records, especially during the earlier years of the study period. Consequently, the numbers of complications presented in this report must be viewed as minimums. A number of patients (n=51) in our catchment area underwent treatment at other institutions and were excluded from this study. In the excluded group, the 90-day mortality and the five-year relative survival rate among those that survived the first 90 days were nearly identical to those observed in the cohort included in this study. However, the addition of these 51 patients to our study cohort might have altered some of the results.

### Future perspectives

The number of older patients with rectal cancer is predicted to escalate in the years to come. This escalation will increase the burden on healthcare systems, at both the national and global levels. Improvements in selecting and treating older patients with rectal cancer might enhance results and optimize the utilization of healthcare resources.

Prehabilitation is gaining interest in the surgical milieu and aims to enhance the individual patient’s starting point prior to surgery. Currently, studies have been investigating the potential of prehabilitation in patients with rectal cancer, and the results may impact the future treatment of older patients [57, 58].

Studies on the effect of age on morbidity have produced conflicting results [59, 60]. Our observation that more severe complications occurred with increasing age may partly be explained by a higher proportion of frail patients in the oldest age groups compared to younger age groups. Frailty may be present in the absence of co-morbid conditions, and it could be a factor in 25-46% of patients over 65 years old that undergo surgery for colorectal cancer [61]. The impact of frailty in patients undergoing surgery for colorectal cancer has been investigated, and it should be emphasized in future clinical practice [62, 63]. Due to the increasing proportion of older patients with rectal cancer, we believe that a comprehensive geriatric assessment should be included as part of the routine work-up.

Physicians may be forced to reconsider treatment aims in older patients, because this group of patients is likely to choose functional status above survival [64]. This choice interferes with one of the most fundamental principles in treating patients with cancer. Moreover, as patients approach the end of life, their personal preferences regarding medical treatment might be more decisive than ever before.

## Conclusion

This study showed that patients aged ≥80 years were less likely to undergo a major resection with curative intent compared to younger patients, despite comparable disease stages. The rate of complications following rectal cancer surgery was high across all ages, but the severity of complications increased with age. Patients aged ≥80 years that underwent a major resection with curative intent had long-term survival rates comparable to their younger counterparts. The future care of older patients with rectal cancer demands highly specialized teams that can focus on the distinctive demands in this specific group of patients.

## Data Availability

The dataset used for this study is located on a secure server in the Levanger Hospital data system. The dataset was confirmed by comparing data with corresponding data in the Norwegian Cancer Registry 1980-2016 (https://www.kreftregisteret.no). The dataset generated and analysed during the current study are not publicly available as their containing information could compromise the privacy of research participants, but are available from the corresponding author on a reasonable request.

## Appendix

Table 9.

**Table 9:** Characteristics of 51 patients diagnosed with rectal cancer during 1980-2016 that were referred to other hospitals for treatment

Characteristic	Total	1980-1989	1990-1999	2000-2009	2010-2016
Age group, years					
<65	25 (49)	0 (0)	0 (0)	10 (59)	15 (47)
65-80	22 (43)	0 (0)	1 (50)	6 (35)	15 (47)
80+	4 (8)	0 (0)	1 (50)	1 (6)	2 (6)
Charlson Comorbidity Index					
0	40 (78)	0 (0)	2 (100)	15 (88)	23 (72)
1	5 (10)	0 (0)	0 (0)	0 (0)	5 (16)
2+	6 (12)	0 (0)	0 (0)	2 (12)	4 (13)
ASA score					
1-2	40 (78)	0 (0)	0 (0)	13 (76)	27 (84)
3	9 (18)	0 (0)	1 (50)	4 (24)	4 (13)
4-5	2 (4)	0 (0)	1 (50)	0 (0)	1 (3)
Localization (Distance from anal verge)					
Proximal (12-15 cm)	10 (20)	0 (0)	0 (0)	3 (18)	7 (22)
Middle (6-11 cm)	20 (39)	0 (0)	2 (100)	9 (53)	9 (28)
Distal (0-5 cm)	21 (41)	0 (0)	0 (0)	5 (29)	16 (50)
Stage (TNM)					
I	13 (25)	0 (0)	0 (0)	5 (29)	8 (25)
II	16 (31)	0 (0)	0 (0)	6 (35)	10 (31)
III	7 (14)	0 (0)	0 (0)	2 (12)	5 (16)
IV	13 (25)	0 (0)	0 (0)	4 (23)	9 (28)
Unknown	2 (4)	0 (0)	2 (100)	0 (0)	0 (0)
Treatment intent categories					
Curative intent					
Major resection	33	0 (0)	0 (0)	10 (63)	23 (78)
Polypectomy	4	0 (0)	2 (100)	2 (13)	0 (0)
Non-curative intent					
Major resection	8	0 (0)	0 (0)	2 (13)	6 (20)
Bypass/Stoma	1	0 (0)	0 (0)	0 (0)	1 (3)
Best supportive care	2	0 (0)	0 (0)	2 (13)	0 (0)

Four patients (7.8%) died within 100 days. For the remaining patients, the 5-year relative survival was 67.1% (95% CI: 49.5-81.2)

## Abbreviations

APR: Abdominoperineal resection
ASA: American Society of Anaesthesiology
CCI: Charlson Comorbidity Index
CD: Clavien-Dindo
CI: Confidence interval
HP: Hartmann's procedure
LARS: Low anterior resection syndrome
MDT: Multidisciplinary team
OR: Odds ratio
SD: Standard deviation
TME: Total mesorectal excision

## References

[1] Cancer Registry of Norway. Cancer in Norway 2020 - Cancer incidence, mortality, survival and prevalence in Norway. Oslo: Cancer Registry of Norway, 2021. Available at: https://www.kreftregisteret.no/globalassets/cancer-in-norway/2020/cin-2020.pdf Accessed 23 June 2022.

[2] Helsedirektoratet. Nasjonalt handlingsprogram med retningslinjer for diagnostikk, behandling og oppfølging av kreft i tykktarm og endetarm. (Norwegian) [National action program with guidelines for diagnosis, treatment and follow-up of cancer of the colon and rectum]. 2019. Available at: https://www.helsedirektoratet.no/retningslinjer/kreft-i-tykktarm-og-endetarm-handlingsprogram/Nasjonalt%20handlingsprogram%20kreft%20i%20tykktarm%20og%20endetarm.pdf/_/attachment/inline/15a3b670-d1eb-454c-b233-a43b7d636694:a187c33ef51e5e08a3890bd25c99fba242341aa3/Nasjonalt%20handlingsprogram%20kreft%20i%20tykktarm%20og%20endetarm.pdf Accessed June 23, 2022.

[3] Heald RJ, Husband EM, Ryall RD (1982). The mesorectum in rectal cancer surgery–the clue to pelvic recurrence?. Br J Surg.

[4] Jullumstro E, Wibe A, Lydersen S, Edna TH (2012). Violation of treatment guidelines – hazard for rectal cancer patients. Int J Colorectal Dis.

[5] Nygren J, Thacker J, Carli F, Fearon KC, Norderval S, Lobo DN (2012). Guidelines for perioperative care in elective rectal/pelvic surgery: Enhanced Recovery After Surgery (ERAS®) Society recommendations. Clin Nutr.

[6] Gustafsson UO, Scott MJ, Schwenk W, Demartines N, Roulin D, Francis N (2013). Guidelines for perioperative care in elective colonic surgery: Enhanced Recovery After Surgery (ERAS®) Society recommendations. World J Surg.

[7] Bjerkeset T, Edna TH (1996). Rectal cancer: The influence of type of operation on local recurrence and survival. Eur J Surg.

[8] Weerink LBM, Gant CM, van Leeuwen BL, de Bock GH, Kouwenhoven EA, Faneyte IF (2018). Long-term survival in octogenarians after surgical treatment for colorectal cancer: Prevention of postoperative complications is key. Ann Surg Oncol.

[9] Hoydahl O, Edna TH, Xanthoulis A, Lydersen S, Endreseth BH (2020). Long-term trends in colorectal cancer: Incidence, localization, and presentation. BMC Cancer.

[10] Sobin LH, Wittekind C (2002). TNM Classification of Malignant Tumours.

[11] Heald RJ, Moran BJ, Ryall RD, Sexton R, MacFarlane JK (1998). Rectal cancer: the Basingstoke experience of total mesorectal excision, 1978–1997. Arch Surg.

[12] Guren MG, Korner H, Pfeffer F, Myklebust TA, Eriksen MT, Edna TH (2015). Nationwide improvement of rectal cancer treatment outcomes in Norway, 1993–2010. Acta Oncol.

[13] American Society of Anesthesiologists. ASA Physical Status Classification System. Available at: https://www.asahq.org/standards-and-guidelines/asa-physical-status-classification-system Accessed 24 June 2022

[14] Charlson ME, Pompei P, Ales KL, MacKenzie CR (1987). A new method of classifying prognostic comorbidity in longitudinal studies: Development and validation. J Chronic Dis.

[15] Dindo D, Demartines N, Clavien PA (2004). Classification of surgical complications: A new proposal with evaluation in a cohort of 6336 patients and results of a survey. Ann Surg.

[16] Coviello PWDE (2015). Estimating and modeling relative survival. The Stata Journal.

[17] The Human Mortality Database. Available at: http://www.mortality.org Accessed 24 June 2022.

[18] Arnold M, Sierra MS, Laversanne M, Soerjomataram I, Jemal A, Bray F (2017). Global patterns and trends in colorectal cancer incidence and mortality. Gut.

[19] Sonstebo A. Vi blir stadig eldre. (Norwegian) [We are getting older]. Statistics Norway 2020. Available at: https://www.ssb.no/befolkning/artikler-og-publikasjoner/vi-blir-stadig-eldre Accessed 24 June 2022

[20] Bertagnolli MM, Singh H (2021). Treatment of older adults with cancer - Addressing gaps in evidence. N Engl J Med.

[21] Chang GJ, Skibber JM, Feig BW, Rodriguez-Bigas M (2007). Are we undertreating rectal cancer in the elderly?. An epidemiologic study. Ann Surg.

[22] Benitez Majano S, Di Girolamo C, Rachet B, Maringe C, Guren MG, Glimelius B (2019). Surgical treatment and survival from colorectal cancer in Denmark, England, Norway, and Sweden: A population-based study. Lancet Oncol.

[23] Endreseth  BH, Romundstad  P, Myrvold  HE, Bjerkeset  T, Wibe  A (2006). Norwegian Rectal Cancer Group. Rectal cancer treatment of the elderly. Colorectal Dis.

[24] Boakye D, Rillmann B, Walter V, Jansen L, Hoffmeister M, Brenner H (2018). Impact of comorbidity and frailty on prognosis in colorectal cancer patients: A systematic review and meta-analysis. Cancer Treat Rev.

[25] Hamaker ME, Rostoft S (2021). Geriatric assessment in older patients with cancer: A new standard of care. Lancet.

[26] Rutten HJ, den Dulk M, Lemmens VE, van de Velde CJ, Marijnen CA (2008). Controversies of total mesorectal excision for rectal cancer in elderly patients. Lancet Oncol.

[27] Kim JH (2015). Chemotherapy for colorectal cancer in the elderly. World J Gastroenterol.

[28] Venderbosch S, Doornebal J, Teerenstra S, Lemmens W, Punt CJ, Koopman M (2012). Outcome of first line systemic treatment in elderly compared to younger patients with metastatic colorectal cancer: A retrospective analysis of the CAIRO and CAIRO2 studies of the Dutch Colorectal Cancer Group (DCCG). Acta Oncol.

[29] Cancer Registry of Norway. Cancer in Norway 2019 - Cancer incidence, mortality, survival and prevalence in Norway. Oslo: Cancer Registry of Norway, 2020. Available at: https://www.kreftregisteret.no/globalassets/cancer-in-norway/2019/cin_report.pdf Accessed 24 June 2022

[30] Brouwer NPM, Bos A, Lemmens V, Tanis PJ, Hugen N, Nagtegaal ID (2018). An overview of 25 years of incidence, treatment and outcome of colorectal cancer patients. Int J Cancer.

[31] Danckert BF, Engholm G, Hansen HL, Johannesen T, Khan S, Køtlum J, et al. NORDCAN: Cancer Incidence, Mortality, Prevalence and Survival in the Nordic Countries, Version 8.2 (26.03.2019) Association of the Nordic Cancer Registries. Danish Cancer Society. Available at: http://www.ancr.nu Accessed 24 June 2022

[32] Stornes T, Wibe A, Romundstad PR, Endreseth BH (2014). Outcomes of rectal cancer treatment–influence of age?. Int J Colorectal Dis.

[33] Jung B, Pahlman L, Johansson R, Nilsson E (2009). Rectal cancer treatment and outcome in the elderly: An audit based on the Swedish Rectal Cancer Registry 1995–2004. BMC Cancer.

[34] Peltrini R, Imperatore N, Carannante F, Cuccurullo D, Capolupo GT, Bracale U, Caricato M, Corcione F. Updates Surg. 2021;73(2):527–37. 10.1007/s13304-021-00990-z. 10.1007/s13304-021-00990-z PMC8005386 33586089

[35] Sverrisson I, Nikberg M, Chabok A, Smedh K. Hartmann’s procedure in rectal cancer: A population-based study of postoperative complications. Int J Colorectal Dis. 2015;30(2):181–6. 10.1007/s00384-014-2069-6. 10.1007/s00384-014-2069-6 25421100

[36] Jonker FH, Tanis PJ, Coene PP, Gietelink L, van der Harst E, Dutch Surgical Colorectal Audit Group. Comparison of a low Hartmann's procedure with low colorectal anastomosis with and without defunctioning ileostomy after radiotherapy for rectal cancer: Results from a national registry. Colorectal Dis 2016;18(8):785–92. 10.1111/codi.13281. 10.1111/codi.13281 26788679

[37] Pieniowski EHA, Nordenvall C, Palmer G, Johar A, Tumlin Ekelund S, Lagergren P (2020). Prevalence of low anterior resection syndrome and impact on quality of life after rectal cancer surgery: Population-based study. BJS Open.

[38] Malik T, Lee MJ, Harikrishnan AB (2018). The incidence of stoma related morbidity - a systematic review of randomised controlled trials. Ann R Coll Surg Engl.

[39] Pahlman L, Bohe M, Cedermark B, Dahlberg M, Lindmark G, Sjodahl R (2007). The Swedish rectal cancer registry. Br J Surg.

[40] Adams WJ, Mann LJ, Bokey EL, Chapuis PH, Koorey SG, Hughes WJ. Hartmann’s procedure for carcinoma of the rectum and sigmoid colon. Aust N Z J Surg. 1992;62(3):200–3. 10.1111/j.1445-2197.1992.tb05463.x. 10.1111/j.1445-2197.1992.tb05463.x 1372498

[41] Paun BC, Cassie S, MacLean AR, Dixon E, Buie WD (2010). Postoperative complications following surgery for rectal cancer. Ann Surg.

[42] Singh J, Stift A, Brus S, Kosma K, Mittlbock M, Riss S (2014). Rectal cancer surgery in older people does not increase postoperative complications–a retrospective analysis. World J Surg Oncol.

[43] Stornes T, Wibe A, Endreseth BH (2016). Complications and risk prediction in treatment of elderly patients with rectal cancer. Int J Colorectal Dis.

[44] Wren SM, Martin M, Yoon JK, Bech F (2010). Postoperative pneumonia-prevention program for the inpatient surgical ward. J Am Coll Surg.

[45] Burns EM, Bottle A, Aylin P, Darzi A, Nicholls RJ, Faiz O (2011). Variation in reoperation after colorectal surgery in England as an indicator of surgical performance: Retrospective analysis of hospital episode statistics. BMJ.

[46] Siragusa L, Sensi B, Vinci D, Franceschilli M, Pathirannehalage DC, Bagaglini G (2021). Volume-outcome relationship in rectal cancer surgery. Discov Oncol.

[47] Burns EM, Bottle A, Almoudaris AM, Mamidanna R, Aylin P, Darzi A (2013). Hierarchical multilevel analysis of increased caseload volume and postoperative outcome after elective colorectal surgery. Br J Surg.

[48] Youl P, Philpot S, Theile DE, for Cancer Alliance Queensland. Outcomes after rectal cancer surgery: A population-based study using quality indicators. J Healthc Qual 2019;41(6):e90–100. 10.1097/JHQ.0000000000000200. 10.1097/JHQ.0000000000000200 31135608

[49] Makela JT, Klintrup KH, Rautio TT (2017). Mortality and survival after surgical treatment of colorectal cancer in patients aged over 80 years. Gastrointest Tumors.

[50] Kreftregisteret. Nasjonalt kvalitetsregister for tykk- og endetarmskreft, Årsrapport 2020. (Norwegian) [National quality register for colorectal cancer, Annual report, 2020]. Cancer Registry of Norway, 2021.

[51] Barrier A, Ferro L, Houry S, Lacaine F, Huguier M (2003). Rectal cancer surgery in patients more than 80 years of age. Am J Surg.

[52] Mollen RM, Damhuis RA, Coebergh JW (1997). Local recurrence and survival in patients with rectal cancer, diagnosed 1981–86: A community hospital-based study in the south-east Netherlands. Eur J Surg Oncol.

[53] Kiran RP, Pokala N, Dudrick SJ (2007). Long-term outcome after operative intervention for rectal cancer in patients aged over 80 years: Analysis of 9,501 patients. Dis Colon Rectum.

[54] Jullumstro E, Wibe A, Lydersen S, Edna TH (2011). Colon cancer incidence, presentation, treatment and outcomes over 25 years. Colorectal Dis.

[55] Kreftregisteret. Nasjonalt kvalitetsregister for tykk- og endetarmskreft. Årsrapport 2015. (Norwegian) [National quality register for colorectal cancer, Annual report 2015], Cancer registry of Norway, 2016.

[56] Krokstad S, Langhammer A, Hveem K, Holmen TL, Midthjell K, Stene TR (2013). Cohort profile: The HUNT Study. Norway. Int J Epidemiol.

[57] van Rooijen S, Carli F, Dalton S, Thomas G, Bojesen R, Le Guen M (2019). Multimodal prehabilitation in colorectal cancer patients to improve functional capacity and reduce postoperative complications: The first international randomized controlled trial for multimodal prehabilitation. BMC Cancer.

[58] Moug SJ, Mutrie N, Barry SJE, Mackay G, Steele RJC, Boachie C (2019). Prehabilitation is feasible in patients with rectal cancer undergoing neoadjuvant chemoradiotherapy and may minimize physical deterioration: Results from the REx trial. Colorectal Dis.

[59] Shahir MA, Lemmens VE, van de Poll-Franse LV, Voogd AC, Martijn H, Janssen-Heijnen ML (2006). Elderly patients with rectal cancer have a higher risk of treatment-related complications and a poorer prognosis than younger patients: A population-based study. Eur J Cancer.

[60] Alves A, Panis Y, Mathieu P, Mantion G, Kwiatkowski F, Slim K, et al. Postoperative mortality and morbidity in French patients undergoing colorectal surgery: Results of a prospective multicenter study. Arch Surg 2005;140(3):278–83, discussion 284. 10.1001/archsurg.140.3.278. 10.1001/archsurg.140.3.278 15781793

[61] Fagard K, Leonard S, Deschodt M, Devriendt E, Wolthuis A, Prenen H (2016). The impact of frailty on postoperative outcomes in individuals aged 65 and over undergoing elective surgery for colorectal cancer: A systematic review. J Geriatr Oncol.

[62] Ronning B, Wyller TB, Jordhoy MS, Nesbakken A, Bakka A, Seljeflot I (2014). Frailty indicators and functional status in older patients after colorectal cancer surgery. J Geriatr Oncol.

[63] Kristjansson SR, Nesbakken A, Jordhoy MS, Skovlund E, Audisio RA, Johannessen HO (2010). Comprehensive geriatric assessment can predict complications in elderly patients after elective surgery for colorectal cancer: A prospective observational cohort study. Crit Rev Oncol Hematol.

[64] Festen S, van Twisk YZ, van Munster BC, de Graeff P. “What matters to you?” Health outcome prioritisation in treatment decision-making for older patients. Age Ageing. 2021;50(6):2264–9. 10.1093/ageing/afab160. 10.1093/ageing/afab160 PMC8581373 34343234

